# High-efficiency genomic editing in Epstein-Barr virus-transformed lymphoblastoid B cells using a single-stranded donor oligonucleotide strategy

**DOI:** 10.1038/s42003-019-0559-3

**Published:** 2019-08-14

**Authors:** Andrew D. Johnston, Claudia A. Simões-Pires, Masako Suzuki, John M. Greally

**Affiliations:** Center for Epigenomics and Department of Genetics, Albert Einstein College of Medicine, Bronx, NY 10461 USA

**Keywords:** CRISPR-Cas9 genome editing, CRISPR-Cas9 genome editing, Transcriptional regulatory elements, CRISPR-Cas9 genome editing, Gene regulation

## Abstract

While human lymphoblastoid cell lines represent a valuable resource for population genetic studies, they have usually been regarded as difficult for CRISPR-mediated genomic editing because of very inefficient DNA transfection and retroviral or lentiviral transduction in these cells, which becomes a substantial problem when multiple constructs need to be co-expressed. Here we describe a protocol using a single-stranded donor oligonucleotide strategy for ‘scarless’ editing in lymphoblastoid cells, yielding 12/60 (20%) of clones with homology-directed recombination, when rates of <5–10% are frequently typical for many other cell types. The protocol does not require the use of lentiviruses or stable transfection, permitting lymphoblastoid cell lines to be used for CRISPR-mediated genomic targeting and screening in population genetic studies.

## Abstract (web summary)

Andrew Johnston et al. present a genome-editing protocol for lymphoblastoid cell lines, using a single-stranded donor oligonucleotide strategy for ‘scarless’ editing. This protocol streamlines the genome-editing procedure used for lymphoblastoid cell lines by waiving the need for lentiviruses or stable transfection.

## Introduction

Lymphoblastoid cell lines (LCLs) are generated by immortalizing B lymphocytes in vitro with the lymphotropic Epstein-Barr virus. LCLs have been collected for decades as a self-renewing cellular resource from different individuals, initially focusing on multi-generational families to facilitate linkage mapping^1^, and more recently as a resource for population genetics studies, by the HapMap Consortium^2^, the GEUVADIS Project^3^, and the 1000 Genomes Project^4^, among others. LCL panels have also proven useful in pharmacogenomic studies^5^. Furthermore, individual LCLs have served as reference cell lines for a number of purposes. The 17 member, 3 generation CEPH pedigree 1463 has been the target of Illumina’s Platinum Genomes sequencing, while the mother in the second generation of this family is the B lymphocyte donor for the GM12878 cell line, a tier 1 ENCODE cell line^6^ used in many novel assays, such as those studying chromatin looping^7^ and a DNA methylation assay that we developed^8^, as well as the Genome in a Bottle initiative^9^.

Being able to screen LCLs using the CRISPR system is therefore an obvious next step in understanding the function of genomic variants. CRISPR is used to target the CRISPR associated protein 9 (Cas9) nuclease to a locus to create a double stranded break in the DNA. This can be repaired by non-homologous end joining (NHEJ), resulting in an unpredictable range of mutations, but if the locus is targeted with the simultaneous introduction of a single-stranded donor oligonucleotide (ssODN), this promotes homology-directed repair (HDR) using this DNA sequence. If the ssODN is not completely identical to the repaired sequence but carries a specific sequence variant, the HDR should result in the introduction of that sequence change at the locus. This approach has been used to test the effects of specific variants of interest. By introducing the same variant into cells with different genetic backgrounds, epistatic effects can be identified. The wide range of diversity of genetic backgrounds in existing LCLs makes them suitable for such screens. However, LCLs have rarely been used for genomic editing cells unless lentiviral transfection is used to express Cas9 robustly^10^. Testing the functional variant in a surrogate cell type may be uninformative if the variant is very LCL-specific in its activity. Furthermore, if LCLs are to be used in CRISPR screens of the genome^11^, an effective protocol for its use in this cell type is needed.

The most successful reported use to date of CRISPR in LCLs that did not use lentiviruses does not describe the details or efficiency of their strategy, making their protocol difficult to replicate^12^. For a project described in a companion publication^13^, we needed to edit a multifunctional variant in LCLs. We therefore tested how efficiently homology-directed recombination (HDR) using a single-stranded donor oligonucleotide (ssODN) could be used for ‘scarless’, CRISPR-mediated editing in LCLs.

## Results

### CRISPR/Cas9 editing yields a high proportion of successfully edited clones

**Fig. 1:**
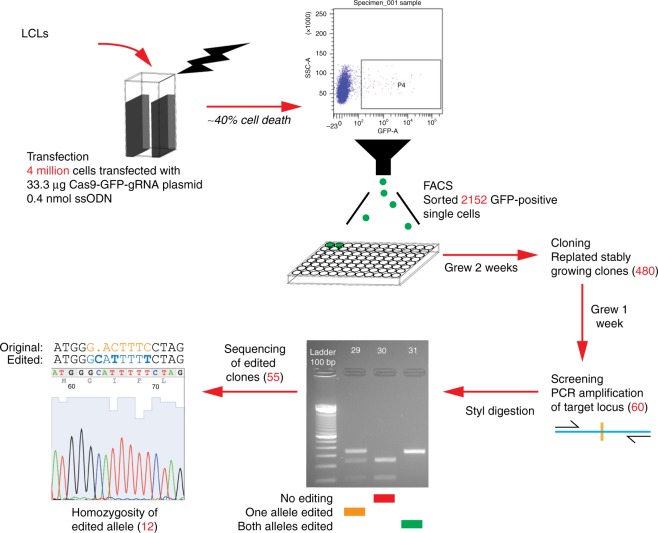
Overview of the LCL editing protocol. We start by transfecting 4 million cells with the plasmid expressing Cas9 and the guide RNA, as well as a green fluorescent protein (GFP) to allow us to sort the transfected cells. The numbers of cells at each stage are shown. Substantial cell death occurs following transfection, but the cell cloning steps were relatively efficient, and the rate of recovery of edited clones was high

**Fig. 2:**
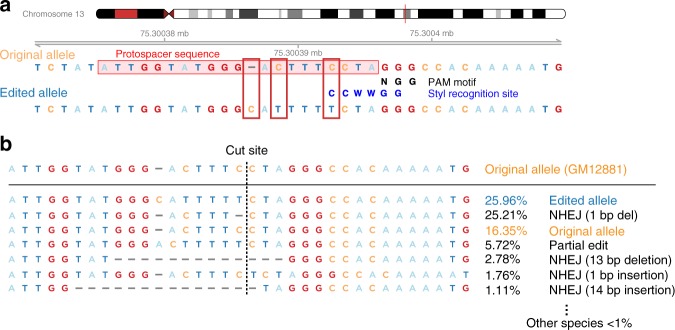
**a** The characteristics of the edited locus are shown. The protospacer sequence, to which the guide RNA binds, is shown to be at the same site as is being targeted for editing by the ssODN, which then prevents the guide RNA from binding to cause further edits. The location of a StyI recognition motif present in the unedited DNA is shown, demonstrating how restriction enzyme digestion with StyI for this particular editing event can be used in screening for editing events. In **b**, we show the results of amplicon sequencing, and the relative frequencies of each type of editing event. The desired editing event is the most common event, followed by non-homologous end joining (NHEJ) with deletion of the single nucleotide immediately at the cut site

Our protocol overview is shown in Fig. 1, the oligonucleotides in Table 1, and the protocol details in the Methods section. Our optimized application of the manufacturer’s transfection settings (Table 2) involved 4 × 10^6^ LCL cells, 33.3 µg of plasmid and 0.4 nmol of ssODN (0.1 nmol/million cells), conditions accompanied by ~40% cell death. GFP was expressed following overnight incubation of transfected cells, achieving a mean 1.45% transfection efficiency (Table 3, Supplementary Fig. 1). Fluorescence-activated cell sorting (FACS) delivered individual GFP+ cells into 96 well plates. We plated over 2000 individual cells, culturing them in conditioned, FBS-rich medium for 2 weeks, resulting in growth of ~22% of clones. We re-plated a total of 480 clones, and chose the 60 fastest-growing clones for screening. Following PCR of the targeted region, we digested the amplicon from each clone with the StyI restriction endonuclease, as its recognition site, present in the unedited cell line, should be disrupted by local genomic editing events (Fig. 2a). This revealed 55 of the 60 clones to have had some sort of editing event at the locus, prompting the sequencing of these amplicons, and revealing that 12 of the 55 had apparent homozygosity for the desired homology-directed recombination (HDR) event. We calculate the HDR rate as 12/60 clones tested, or 20%.

**Table 1:** Primers and oligonucleotides used in this study

Amplicon Primers (1st PCR reaction)	
Amplicon-TBC1D4-FWD-1	ACACTCTTTCCCTACACGACGCTCTTCCGATCTNNCAAGGAAAATAAAGGGTCAAGTCAA
Amplicon-TBC1D4-REV-1	CTGGAGTTCAGACGTGTGCTCTTCCGATCCTTCCTCTTACTGGCTCTGCAG
Amplicon Primers (2nd PCR reaction)	
Amplicon-3A-REV-2	CAAGCAGAAGACGGCATACGAGATCAACAAGTGACTGGAGTTCAGACGT
Amplicon-3A-ssODN-REV-2	CAAGCAGAAGACGGCATACGAGATGTTGTTGTGACTGGAGTTCAGACGT
T7 Endonuclease Primers	
T7-TBC1D4-FWD	GGCCACCATACCATCTTCACA
T7-TBC1D4-REV	ATTTGGCTCTGCTTGTAGCC
RT-PCR 1 kb TBC1D4 amplicons	
TBC1D4_1kb-1-FWD	GCTTTGTGCCAACTAGCATGT
TBC1D4_1kb-1-REV	ACTGGGTAACAGTGCGGAGG
TBC1D4_1kb-2-FWD	AGGGCCAATAGCCAACTGAA
TBC1D4_1kb-2-REV	TGGGTAGACTCAGCCACAATG
TBC1D4_1kb-3-FWD	TGGTGCATGTCAGAAAGAGGT
TBC1D4_1kb-3-REV	TCAGTTGGCTATTGGCCCTC
TBC1D4_1kb-4-FWD	TGAAGCCAAGCAGAAGACACA
TBC1D4_1kb-4-REV	TCCTCGTCGACTTTTGGGAA
TBC1D4_1kb-5-FWD	TTCCAGGTTGGGCGATTTGA
TBC1D4_1kb-5-REV	TCCCTTCTCCATCACTGCAC
4 kb fragment flanking target site	
TBC1D4-4kb-FWD	CCTGGAAAATCTTAATGGTGCTTC
TBC1D4-4kb-REV	GCCTAATAAACAACAGCCTCCTG
TBC1D4 gRNA cloning oligonucleotides and ssODN template (editing)	
TBC1D4 gRNA-edit-FWD	CACC ATTGGTATGGGACTTTCCTA*
TBC1D4 gRNA-edit-REV	AAAC **TAGGAAAGTCCCATACCAAT**
TBC1D4 ssODN Template	CTTGAGCTGGCAATGTGAGTCCTGCATCACTAAAAGGAGAGTTCTATACACAGAAACAAATCCGTCTTCACATCAAAGCTGTCTATATTGGTATGGGCATTTTTCTAGGGCCACAA AAATGAAGGGGGATGTTAGCTTCCTTGTGAAATGATTA CTCATGTCATTTAGAACTTGCAAGAGTGCCAGGTTTTAAAATGTTT

*Sequences in bold correspond to the targeted 20-nt gRNA

**Table 2:** Conditions tested and efficiencies of transfections

Plasmid^a^	Transfection conditions^b^	Number of cells (million)	Plasmid (µg)	Plasmid (molecules)	Approximate transfection efficiency (microscopy, percent GFP + cells)
GFP	X-001	4	2	6.78E + 12	0
	T-020	4	2	6.78E + 12	10–15
	U-009	4	2	6.78E + 12	10–15
pCAG-eCas9-GFP-U6-gRNA	U-009	2	5	4.76E + 11	0
	U-009	4	10	9.51E + 11	1
	U-009	2	16.6	1.58E + 12	0
	U-009	4	33.3	3.17E + 12	2–5

^a^AddGene 79145

^b^Amaxa Nucleofector II programs

**Table 3:** Transfection efficiency testing using fluorescence-activated cell sorting (FACS)

	Total events	LCLs	Single cells (front scatter)	Single cells (side scatter)	GFP positive	% GFP positive
**Replicate 1**	10,000	7965	7629	7493	143	**1.91%**
**Replicate 2**	10,000	7687	7421	7349	94	**1.28%**
**Replicate 3**	10,000	7338	7108	7014	82	**1.17%**
					Mean	**1.45%**
					Standard deviation	**0.40%**

### Amplicon-seq reveals the distribution of different types of editing events

To gain a more detailed insight into the editing events, we performed amplicon sequencing of the pool of cells prior to the cloning step. We show the results of analysis of these data using CRISPResso^14^ in Fig. 2b. The amplicon PCR primers were designed to flank the region homologous with the ssODN sequence. The most common edit was the desired HDR (25.96%), followed by a 1 bp deletion immediately upstream of the cut site representing non-homologous end-joining (NHEJ, 25.21%), with only 16.35% of the sequences representing unedited alleles. In Supplementary Fig. 2, we show that HDR was completely attributable to the presence of the ssODN, and that NHEJ events are most frequent immediately adjacent to the cut site, decreasing in frequency even over 10 bp distance.

### Testing for large rearrangements at the targeted locus

As it has recently been described that CRISPR-mediated genomic editing induces large structural rearrangements, missed when testing the immediate vicinity of the target site^15^, we screened for such events in two ways. We performed long-range PCR of the 4 kb centered on the target site. As this target site is within an intron of a gene transcribed over almost 200 kb of the genome, we also designed RT-PCR primers, each amplifying ~1 kb fragments, spanning multiple exons and thus a larger surrounding region. We tested 5 cell lines with diverse edits (Supplementary Fig. 3a) with the primers in Supplementary Fig. 3b/Table 1. In Supplementary Fig. 3c we show that two clones (one with a 14 bp deletion, the other with a combined edit and indel) amplified PCR products shorter than 4 kb. The RT-PCR amplicons were compared by densitometry and normalized to the signal obtained for the amplification from the 3′ UTR. In Supplementary Fig. 3d we show that for several amplicons the 7 bp deletion and the 14 bp deletion clones are both below the range of densitometric values of the other samples tested. We illustrate in Supplementary Fig. 3b where these indicate there to be larger-scale rearrangements in the clones tested, confirming the need for caution in performing these editing experiments^15^, and demonstrating that the apparent homozygosity observed for the 7 bp deletion clone is instead likely to be due to hemizygosity at this locus.

## Discussion

It has been shown that LCLs can be edited using CRISPR and HDR to change a single nucleotide at a locus regulating local gene expression^12^. We extend this finding to reveal the critical steps in the protocol. We used plasmid and ssODN in proportions found to work for HEK 293 T cells^16^, but increased these in amounts for the higher cell numbers needed to optimize nucleofection. We note that the most critical variable influencing transfection rate was the number of cells used (Table 2), requiring very accurate cell counting be performed. To enhance transfection efficiency, we used published conditions for optimized siRNA transfection in LCLs that involved passaging cells twice to get cells into growth phase before transfection^17^.

Our HDR rate, at 20%, is high relative to results reported from other cell types. We believe that there were a few reasons why this was the case. Our ssODN was designed to avoid hybridizing with the gRNA, given that Cas9 might possibly degrade it^18^. The guide RNA (gRNA) was designed to avoid binding to the template strand transcribed at this intronic location^18^. We also used a double-stranded nuclease instead of nickase^18^. By targeting the HDR-directed mutations to within the guide RNA binding site, we reduce the re-cutting of the site by CRISPR to enhance scarless editing^19^. Potentially the major influence was our introduction using HDR of three sequence changes in close proximity to the cut site, causing the target to become immediately very divergent in sequence to the gRNA, probably substantially reducing the chances of secondary re-editing. As a final possibility, we cannot exclude LCLs being a cell type unusually efficient in performing HDR.

There are some simple guidelines worth following when editing LCLs. The protocol outline shown in Fig. 1 provides some guidance about cell numbers at each step. We had excessive cell loss during culture because the wells at the outside of the multi-well plate evaporated more quickly. A remedy for the future would be to keep those wells filled with water and use only the internal wells for cell culture. Plasmids need to be highly concentrated to avoid diluting nucleofection reagents. We anticipate that the efficiency of the protocol will improve markedly over time. With the introduction of new systems for transfection such as the Neon Transfection System, which Thermo Fisher Scientific claims will yield 80% transfection rates for LCLs, we anticipate that improved transfection will be an early focus for further optimization of our suggested protocol. A further improvement of the protocol is likely to be accomplished with a switch from plasmids to ribonucleoprotein reagents for gRNAs and Cas9. This has been tested in LCLs pre-infected with lentiviruses stably expressing Cas9, demonstrating its feasibility^20^. All of the techniques we used are potentially automated by liquid handling/cell culture systems, raising the possibility that LCL editing may be amenable to scaling to many cell lines at a time, potentially introducing the same variant in multiple genetic backgrounds to create a system for studying phenomena like epistasis, lending further value to this research workhorse cell type.

## Methods

### Cell culture

The lymphoblastoid cell line (LCL) derived from a child within the CEPH Pedigree 1463 (GM12881) was purchased from the Coriell Institute and cultured in RPMI 1640 medium, supplemented with 15% fetal bovine serum (FBS, Gemini Bio-Products, BenchMark grade), 100 IU/ml penicillin, and 100 μg/ml streptomycin (Life Technologies). Cells were kept in suspension in tissue culture flasks (NUNC, Thermo Scientific) at 37 °C in a 5% CO_2_ incubator and maintained between 2 × 10^5^ and 8 × 10^5^ cells/ml.

### Plasmids and ssODN template

Plasmid pCAG-eCas9-GFP-U6-gRNA was a gift from Jizhong Zou (Addgene plasmid #79145) and was used in combination with a single-stranded oligonucleotide (ssODN) template (Table 1) for editing purposes. The 200 nt ssODN was designed using the same approach published by Chiang and colleagues^16^ and was PAGE-purified (Integrated DNA Technologies). Plasmid pmaxGFP (Lonza) was used as a GFP positive control vector. gRNAs were designed using the CRISPOR web interface^21^. To insert the desired gRNA sequence into the CRISPR/Cas9 vector, reverse complement oligonucleotides containing the 20-nt gRNA target sequence (Table 1) were annealed, 5′-phosphorylated and ligated into the linearized vector.

### CRISPR/Cas9 editing and sorting

For transfection, cells were passaged at 3.5 × 10^5^ 48 h and 24 h before transfection. A total of 4 × 10^6^ GM12881 cells were transfected with 33.3 μg of CRISPR/Cas9 plasmid and 0.4 nmol of ssODN template. GFP control cells received 2 μg of GFP plasmid, while negative control cells received transfection reagents only. Transfections were conducted with the Cell Line Nucleofactor Kit V (Lonza) according to the manufacturer’s instructions. After transfection, cells were suspended in medium and incubated overnight under normal cell culture conditions, then replaced with fresh medium. GFP-positive cells were sorted after 48 h following the transfection. The cells were pelleted, washed twice, and suspended in sorting buffer (Hank’s balanced salt solution buffer supplemented with 1% FBS, 100 units/mL penicillin, and 100 μg/mL streptomycin). Cell suspensions were submitted to cell analysis and sorting in a FACSAria II cytometer (BD Biosciences). FACS data were analyzed using FACSDiva software (Becton Dickinson) with gating of single cells using FSC/W and SSC/W, and gating of GFP-positive cells.

### Clone isolation upon genomic editing

Single GFP-positive cells were sorted 48 h after transfection into individual wells of a 96-well plate, containing a mixture of fresh and conditioned medium (1:1), in which the FBS concentration was increased to 20%. The 96-well plate was incubated for two weeks under cell culture conditions, then the clones exhibiting robust growth were transferred to a new 96-well plate with additional conditioned medium. Given the high concentration of FBS and the long culture times, a precipitate may form, which can easily be mistaken for contamination. Driven by evaporation, we recommend plating water in the peripheral wells of a 96-well plate to avoid disruption of cell growth by crystal formation. Conditioned medium was obtained from GM12881 cells, and cultured in 20% FBS RPMI 1640 for 24 h. The medium was removed without disturbing cells at the bottom of the flask, centrifuged at 2000 rpm, and the supernatant was filtered through a 0.3 micron sterile filter prior to use. When subsequent T7EI assays were to be performed, cells were sorted directly into QuickExtract DNA extraction solution (Epicenter).

### T7 endonuclease I assay (T7EI)

To verify editing in clones, in which an intronic enhancer of *TBC1D4* was targeted, genomic DNA was isolated from transfected and control cell pellets using QuickExtract DNA Extraction Solution (Epicenter) according to manufacturer’s instructions. DNA was then concentrated by ethanol precipitation. The 1 kb region containing the gRNA targeted region was amplified with forward and reverse primers (Table 1) using the Q5 Hot Start High-Fidelity 2× Master Mix (NEB) according to the manufacturer’s protocol with 100 ng of the purified total cellular DNA in a 50 μl reaction. Amplification products were isolated using the DNA Clean and Concentrator-5 kit (Zymo Research). PCR product (50 ng) was denatured and re-annealed in a final volume of 13 μl in 1× NEBuffer2 (NEB) using a thermocycler with the following protocol: 95 °C, 5 min; 95→85 °C at −2 °C/s; 85→25 °C at −0.1 °C/s; hold at 4 °C. Hybridized PCR products were treated with 10 U of T7E1 enzyme (NEB) at 37 °C for 60 min in a reaction volume of 20 µL. The reaction was stopped with 2 µL of 0.25 M EDTA, and subsequently analyzed on a 1.5% agarose gel.

### Amplicon-seq generation and data analysis

Cell lines generated by CRISPR/Cas9 editing at a locus with or without a repair template were assessed by amplicon-seq. A suspension of 10^4^ cells sorted in QuickExtract DNA extraction solution (Epicenter) was used for DNA extraction according to manufacturer’s instructions. DNA was extracted by vortexing the cell suspension for 15 s, followed by incubation at 65 °C for 6 min, an additional 15 s vortexing, and a final 2 min incubation at 98 °C. DNA was then concentrated by ethanol precipitation and submitted to an initial PCR with locus-specific forward and reverse primers with portions of the Illumina TruSeq adapters on their 5′ ends. PCR products were purified with DNA Clean and Concentrator-5 kit (Zymo), and a second round of PCR was performed on purified DNA with primers containing the remaining Illumina adapter along with a custom 6-nt index on the reverse primer. The amplicon libraries were then purified by gel extraction and sequencing using Illumina MiSeq technology, 250 bp single end sequencing. The resulting data were analyzed using CRISPResso^14^.

### RT-PCR of the *TBC1D4* 1 kb amplicons

Cell pellets were treated with QIAzol lysis reagent (Qiagen) and total RNA was isolated using the miRNAeasy kit (Qiagen) combined with on column DNAse (Qiagen) treatment according to manufacturer’s instructions. Synthesis of cDNA was performed with total RNA and SuperScript III First-Strand Synthesis System for RT-PCR (Life technologies) using oligo(dT)_20_ as primers. Subsequent PCR was conducted with primers designed using the NCBI Primer-BLAST web interface^22^ (Table 1) and the Q5 hot start high fidelity polymerase master mix, according to the manufacturer’s protocol. Samples were then analyzed on a 2% agarose gel.

### Reporting summary

Further information on research design is available in the Nature Research Reporting Summary linked to this article.

## Supplementary information

Supplementary Information

Reporting Summary

## Data Availability

All genome sequencing data are available from the NCBI Gene Expression Omnibus database under accession number GSE117576.

## Code availability

CRISPResso was used to analyze the amplicon seq libraries (https://github.com/lucapinello/CRISPResso; downloaded 04/04/18). All code used to analyze the data can be found here: https://github.com/AJEinstein/Johnston-LCL-editing. For generation of FACS plots, we used FlowJo v. 10.5.0.
