# Supplementary material for: High-efficiency genomic editing in Epstein-Barr virus-transformed lymphoblastoid B cells using a single-stranded donor oligonucleotide strategy
Source: Commun Biol. 2019 Aug 14;2:312. doi: 10.1038/s42003-019-0559-3 (PMC6694121; doi:10.1038/s42003-019-0559-3)
Supplement: Supplementary file 1 — Supplementary Information [file 42003_2019_559_MOESM1_ESM.docx]

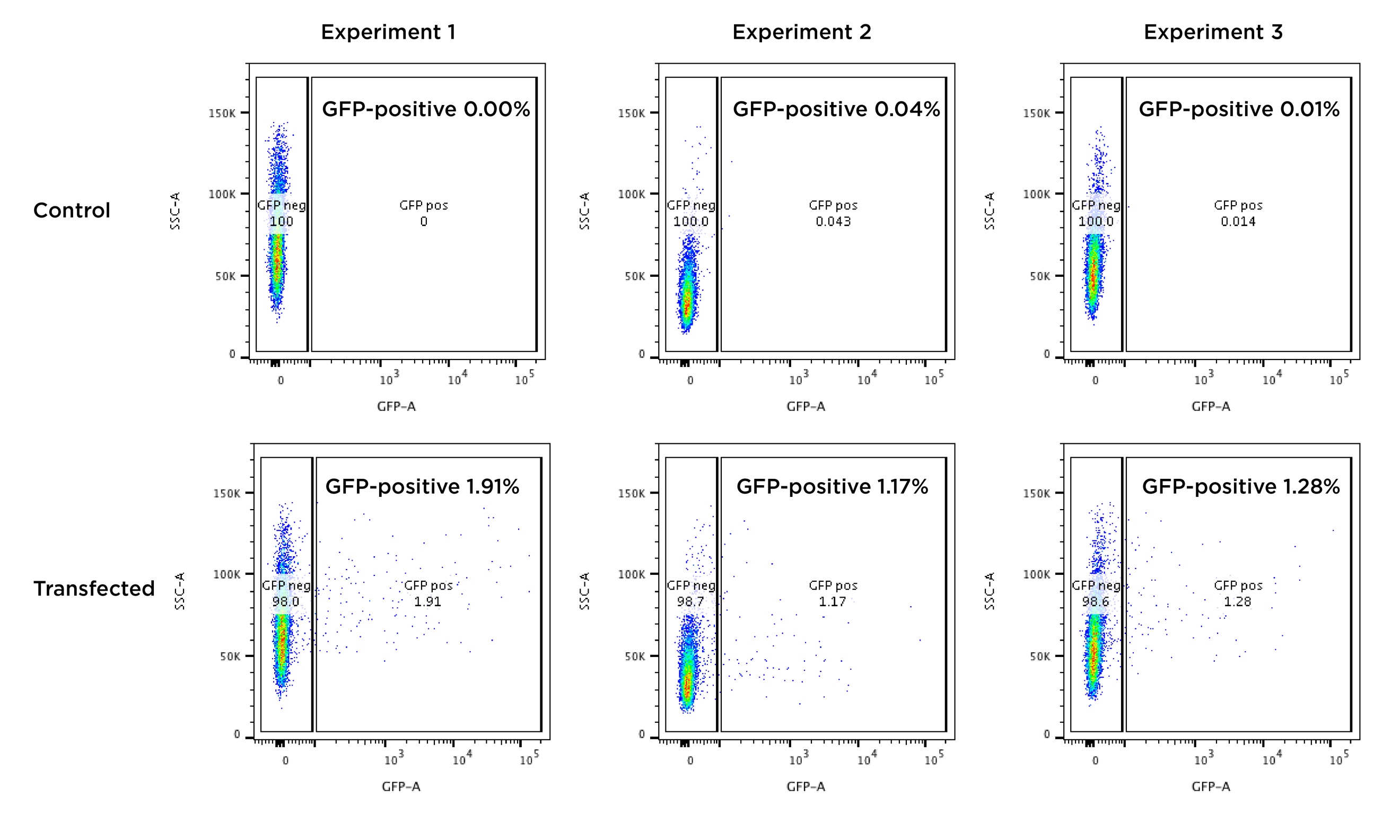


**Supplementary** **Figure 1:** Flow cytometry data. The LCL transfection efficiency averages 1.45% across the three experiments.


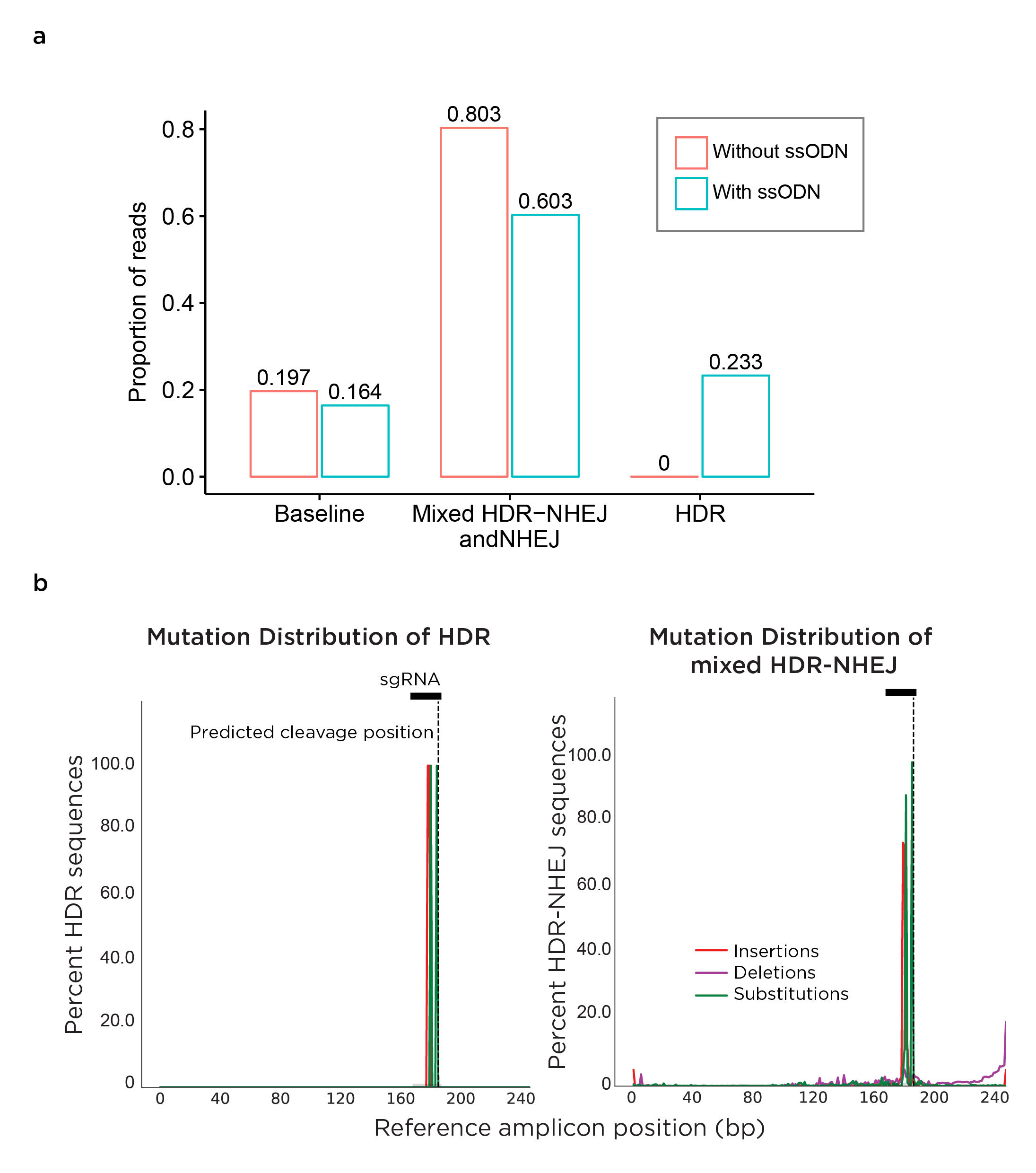


**Supplementary** **Figure 2:** (a) The use of the ssODN is responsible for all HDR events, bringing its frequency from zero to over 23%. In (b) we show the three events in the HDR alleles, allowing us to appreciate what is illustrated on the right, that NHEJ events occur more frequently closer to the cut site and diminish in frequency dramatically over <10 bp.


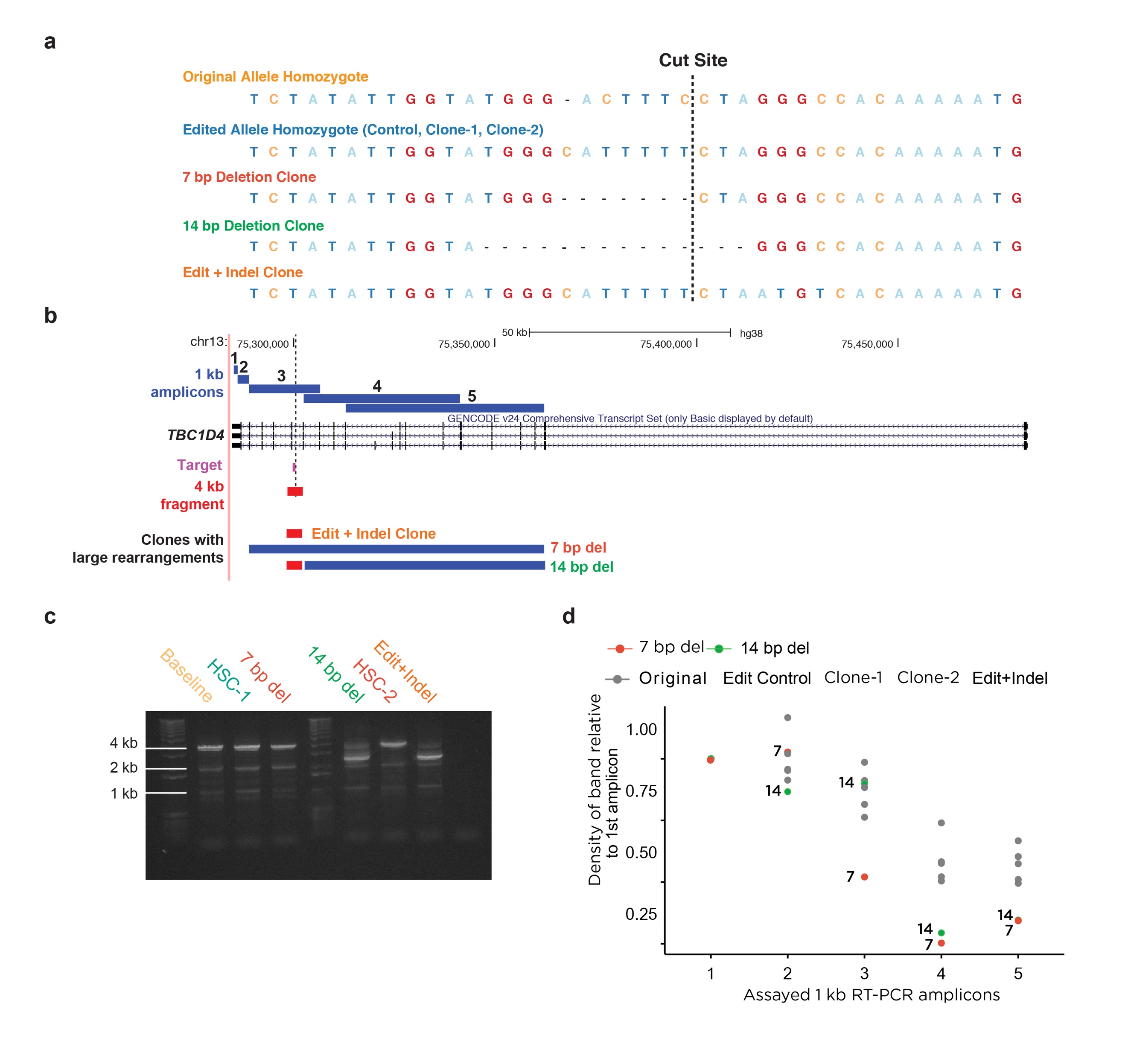


**Supplementary** **Figure 3:** In (a) we show the unedited sequence and the editing events at the target site in 4 different clones. In (b) we show the location of the 4 kb PCR of the DNA flanking the edited region (red), and the five sets of RT-PCR primers amplifying 1 kb fragments of the *TBC1D4* mRNA. In (c) we show the results of the 4 kb DNA PCR, showing two clones (14 bp deletion and the edit/indel clone) to have <4 kb amplicons, indicating that these have deletions within the amplified region. In (d) we show the densitometry of the RT-PCR products normalized to the 3’ UTR signal. The 7 and 14 denote the 7 bp and 14 bp deletion clones, respectively. The range of values shows the 7 bp and 14 bp deletion clones to be outliers, indicating differing sized deletions in each clone. The likely locations of deletions are represented at the bottom of panel (b). The source data underlying this plot are shown in Supplementary Table 1.

**Supplementary Table 1**. Densitometry of 1 kb fragments of TBC1D4 RT-PCR product normalized to first fragment. This table represents the source data underlying the plot shown in supplementary Figure 3d.

| **Fragment** | **Original** | **HSC-1** | **HSC-2** | **7bp-del** | **14bp-del** | **Edit Control** | **Edit+indel** |
| --- | --- | --- | --- | --- | --- | --- | --- |
| 1 kb | 1 | 1 | 1 | 1 | 1 | 1 | 1 |
| 2 kb | 0.96 | 0.95 | 0.91 | 1.03 | 0.87 | 1.02 | 1.17 |
| 3 kb | 0.91 | 0.99 | 0.76 | 0.52 | 0.90 | 0.88 | 0.82 |
| 4 kb | 0.58 | 0.74 | 0.58 | 0.25 | 0.29 | 0.50 | 0.52 |
| 5 kb | 0.67 | 0.50 | 0.51 | 0.35 | 0.35 | 0.60 | 0.57 |
