# Supplementary material for: High-efficiency genomic editing in Epstein-Barr virus-transformed lymphoblastoid B cells using a single-stranded donor oligonucleotide strategy
Source: Commun Biol. 2019 Aug 14;2:312. doi: 10.1038/s42003-019-0559-3 (PMC6694121; doi:10.1038/s42003-019-0559-3)
Supplement: Supplementary file 2 — Reporting Summary [file 42003_2019_559_MOESM2_ESM.pdf]

nature research

Corresponding author(s): John Greally, July 29, 2019

Reporting Summary

Nature Research wishes to improve the reproducibility of the work that we publish. This form provides structure for consistency and transparency in reporting. For further information on Nature Research policies, see Authors & Referees and the Editorial Policy Checklist.

Statistical parameters

When statistical analyses are reported, confirm that the following items are present in the relevant location (e.g. figure legend, table legend, main text, or Methods section).

n/a	Confirmed	
☐	☒	The exact sample size (*n*) for each experimental group/condition, given as a discrete number and unit of measurement
☐	☒	An indication of whether measurements were taken from distinct samples or whether the same sample was measured repeatedly
☒	☐	The statistical test(s) used AND whether they are one- or two-sided<br>*Only common tests should be described solely by name; describe more complex techniques in the Methods section.*
☐	☒	A description of all covariates tested
☒	☐	A description of any assumptions or corrections, such as tests of normality and adjustment for multiple comparisons
☒	☐	A full description of the statistics including central tendency (e.g. means) or other basic estimates (e.g. regression coefficient) AND variation (e.g. standard deviation) or associated estimates of uncertainty (e.g. confidence intervals)
☒	☐	For null hypothesis testing, the test statistic (e.g. *F*, *t*, *r*) with confidence intervals, effect sizes, degrees of freedom and *P* value noted<br>*Give P values as exact values whenever suitable.*
☒	☐	For Bayesian analysis, information on the choice of priors and Markov chain Monte Carlo settings
☒	☐	For hierarchical and complex designs, identification of the appropriate level for tests and full reporting of outcomes
☒	☐	Estimates of effect sizes (e.g. Cohen's *d*, Pearson's *r*), indicating how they were calculated
☒	☐	Clearly defined error bars<br>*State explicitly what error bars represent (e.g. SD, SE, CI)*

Our web collection on statistics for biologists may be useful.

Software and code

Policy information about availability of computer code

Data collection	FACSDiva software (Becton Dickinson) v. 8.0.1
Data analysis	CRISPResso was used to analyze the amplicon seq libraries (https://github.com/lucapinello/CRISPResso; downloaded 04/04/18). All code used to analyze the data can be found here: https://github.com/AJEinstein/Johnston-LCL-editing. For generation of FACS plots, we used FlowJo v. 10.5.0.

For manuscripts utilizing custom algorithms or software that are central to the research but not yet described in published literature, software must be made available to editors/reviewers upon request. We strongly encourage code deposition in a community repository (e.g. GitHub). See the Nature Research guidelines for submitting code & software for further information.

Data

Policy information about availability of data

All manuscripts must include a data availability statement. This statement should provide the following information, where applicable:

- Accession codes, unique identifiers, or web links for publicly available datasets
- A list of figures that have associated raw data
- A description of any restrictions on data availability

All genome sequencing data are available from the NCBI Sequence Read Archive under accession number SRP155069 (https://trace.ncbi.nlm.nih.gov/Traces/sra/sra.cgi?study=SRP155069). Processed sequencing data was used to generated Figure 2B.

Field-specific reporting

Please select the best fit for your research. If you are not sure, read the appropriate sections before making your selection.

☒ Life sciences ☐ Behavioural & social sciences ☐ Ecological, evolutionary & environmental sciences

For a reference copy of the document with all sections, see nature.com/authors/policies/ReportingSummary-flat.pdf

Life sciences study design

All studies must disclose on these points even when the disclosure is negative.

Sample size	We repeated the tranfection protocol three times, once for clonal characterization and twice for amplicon-seq libraries, to ensure that the results were reproducible. The results from the replicate amplicon-seq can be found here: https://github.com/AJEinstein/Johnston-LCL-editing.
Data exclusions	No generated data was excluded.
Replication	We further characterized clones showing several phenotypes through qRT-PCR of the overlying gene as well as amplification of the surrounding 4 kb amplicon. This internal replication gave us an idea as to the clones true genotypes and is presented in Figure S3.
Randomization	The successfully edited clones were chosen at random; however, randomization was irrelevant to the study design as the observers had no a priori knowledge as to the GFP+ cells that represented successfully edited cells.
Blinding	Blinding is irrelevant for the cell lines as they should not demonstrate treatment bias. Blinding of observers was not possible.

Reporting for specific materials, systems and methods

Materials & experimental systems

n/a	Involved in the study	
☒	☐	Unique biological materials
☒	☐	Antibodies
☐	☒	Eukaryotic cell lines
☒	☐	Palaeontology
☒	☐	Animals and other organisms
☒	☐	Human research participants

Methods

n/a	Involved in the study	
☒	☐	ChIP-seq
☐	☒	Flow cytometry
☒	☐	MRI-based neuroimaging

Eukaryotic cell lines

Policy information about cell lines

Cell line source(s)	Coriell Institute
Authentication	We were able to compare RNA-seq, sRNA-seq, DNA bisulphite-seq, and ATACseq from the cell lines used in this study to the DNA-seq results from the Illumina Platinum Genomes Project. We verified that the cell lines positively matched those used in the Illumina Platinum Genomes Project using the QTLtools --mbv function (https://qtltools.github.io/qtltools/; see results and code here https://github.com/GreallyLab/Johnston_et_al/tree/master/Genotype_Library_Match)

Mycoplasma contamination	While Coriell certifies that the cell lines are Mycoplasma free, mycoplasma testing was not performed upon or after receipt.
Commonly misidentified lines<br>(See ICLAC register)	N/A

Flow Cytometry

Plots

Confirm that:

☒ The axis labels state the marker and fluorochrome used (e.g. CD4-FITC).

☒ The axis scales are clearly visible. Include numbers along axes only for bottom left plot of group (a 'group' is an analysis of identical markers).

☒ All plots are contour plots with outliers or pseudocolor plots.

☒ A numerical value for number of cells or percentage (with statistics) is provided.

Methodology

Sample preparation	Briefly, cells were pelleted, washed twice, and resuspended in sorting buffer (Hank's balanced salt solution buffer supplemented with 1% FBS, 100 units/mL penicillin, and 100 mg/mL streptomycin).
Instrument	FACSAria II cytometer (BD Biosciences)
Software	FACSDiva software (Becton Dickinson)
Cell population abundance	On average, we successfully transfected 1.45% of lymphoblastoid cell lines with our plasmid. We performed amplicon-seq of our CRISPR targeted locus and believe that the post-sort fraction was pure because the overall efficiency of CRISPR edited alleles was high (80-85%).
Gating strategy	We used cells transfected without plasmid as controls to set a stringent gating strategy (10^2 GFP-A), in which only GFP+ cells would be collected. The degree of GFP fluorescence varied within our treated cells, and we believe our cutoff to be conservative, as to only accurately describe the genetic populations within treated cells.

☒ Tick this box to confirm that a figure exemplifying the gating strategy is provided in the Supplementary Information.
